# Cervical cancer: a qualitative study on subjectivity, family, gender and health services

**DOI:** 10.1186/1742-4755-4-2

**Published:** 2007-03-01

**Authors:** Blanca E Pelcastre-Villafuerte, Laura L Tirado-Gómez, Alejandro Mohar-Betancourt, Malaquías López-Cervantes

**Affiliations:** 1 Centro de Investigación en Sistemas de Salud, Instituto Nacional de Salud Pública, Secretaría de Salud, México, Av, Universidad 655, Santa María Ahuacatitlán, C.P. 62508, Cuernavaca, Morelos, México; 2 Departamento de Epidemiología, Instituto Nacional de Cancerología de México, Av. San Fernando No. 22; 2°, Piso de investigación, Col. Sección XVI., Del Tlalpan, C.P. 01480, México, D.F., México; 3 Dirección General, Instituto Nacional de Cancerología de México, Av. San Fernando No. 22; 3er, Piso de edificio de quimioterapia, Col. Sección XVI., Del Tlalpan, C.P. 01480, México, D.F., México; 4 Facultad de Medicina, Universidad Nacional Autónoma de México, Ciudad Universitaria, Edificio B 3er. Piso, Del Coyoacan C.P. 04510, México, D.F., México

## Abstract

**Background:**

In 2002, cervical cancer was one of the leading causes of death in Mexico. Quantitative techniques allowed for the identification of socioeconomic, behavioral and biological characteristics that are part of its etiology.

However such characteristics, are inadequate to explain sufficiently the role that emotions, family networks and socially-constructed categories such as gender play in the demand and utilization of health services for cervical cancer diagnosis and treatment and neither the timely undertaking of preventive actions, such as getting a PAP smear or seeking adequate and continuons treatment.

**Methods:**

A qualitative study was carried out to analyze the role of different social and cultural factors in the timely detection of cervical cancer. As part of a multi-level, multi-method research effort, this particular study was based on individual interviews with women diagnosed with cervical cancer (identified as the "cases"), their female friends and relatives (identified as the "controls") and the cases' husbands.

**Results:**

The results showed that both: denial and fear are two important components that regulate the behavior of both the women and their partners. Women with a small support network may have limited opportunities for taking action in favor of their own health and wellbeing.

**Conclusion:**

Women tend not to worry about their health, in general and neither about cervical cancer in particular, as a consequence of their conceptualizations regarding their body and feminine identify – both of which are socially determined. Furthermore, it is necessary to improve the quality of information provided in health services.

## Background

In 2002, malignant tumors were the first cause of death among the Mexican working-age population and cervical cancer was the most frequent tumor producing a total of 2,958 deaths, with a rate of 4.9 per 100,000 inhabitants aged 15–64 years [1].

In 1995, most new cases of cervical cancer occurred in the State of Veracruz (10.2%), followed by State of Mexico (8.9%) and Mexico City (8.8%), which has a high population density. The states with the highest mortality rates per 100,000 inhabitants were: Nayarit (9.0), Colima (7.6), Zacatecas (7.2) Morelos (6.8) and Veracruz (6.4) [2].

There are multiple factors involved in cervical cancer etiology, such as: low socioeconomic level, early age at first intercourse, multiple sexual partners, early age at first pregnancy, multiparity, use of hormonal contraceptives and smoking [3-7]. Another factor recently studied as a cervical cancer initiator is the presence of cervical infections, mainly those caused by human papillomavirus (HPV) [8-12].

Therefore, cervical cancer has been approached from different study perspectives: at the molecular level, focusing on the search for and the identification of human papillomavirus; at the cellular level, looking for malignant transformation by means of the Papanicolaou test (PAP); and at the individual level, in search of physical, psychological and familial consequences of the disease.

Quantitative techniques have traditionally been used to improve our knowledge about the natural history of this disease. However, these techniques do not allow to explore the individual and group contexts which, in theory, determine the demand and successful utilization of preventive measures, such as the PAP test.

At the national level, only a few studies provide information about the barriers that limit the effectiveness of programs for the timely detection of cervical cancer. To the best of our knowledge, there is not a single program aimed at increasing male participation in reproductive health matters. The lack of male involvement in early cervical cancer detection programs is a result of the man's position within the couple, and of their ignorance on the consequences of their own attitudes and of their perceptions on this or other diseases [13-15].

The present study attempts to analyze the role of several social and cultural factors in relation to the early detection of cervical cancer, focusing on the influence of the partner and the social networks regarding the utilization of the PAP test [16,17].

## Methods

This work is part of a larger multilevel study which collected information from the molecular (HPV), the cellular and the individual levels (sociodemographic and economic aspects, gyneco-obstetric backgrounds and tobacco habits), and is based on the interpretation of qualitative results from in depth interviews nested within a case-control study.

The purpose of the qualitative section was to identify individual and family characteristics that favour the demand and successful utilization of the PAP test, as well as to identify the barriers that work against a its successful use, by comparing cases of cervical cancer and controls (we incorporated testimonies of women's partners in the analysis). We also compared aspects related to perception and knowledge of the disease, and aspects related to health care access, its effectiveness, and the quality of the program.

For the case-control study, all newly dignosed cases of cervical cancer seeking case at the National Institute of Cancer in Mexico City and the dysplasia clinics in the State of Veracruz were enrolled in the study, conditional upon being residents of Mexico city or the states of Mexico and Veracruz.

Personal interviews were conducted by trained personnel using a guide of items grouped in four sections: i) subjectivity, ii) family, iii) gender, and iv) health services.

At total of 130 cases, 200 controls and 20 husbands of cases were interviewed. For the purpose of this paper, the qualitative analysis was made with a sub-sample of 20 cases (mean age = 45 years), 20 controls (mean age = 42 years) and 11 husbands. A random sample of cases and controls (and husbands) was chosen as soon as we had histological confirmation of the health status. We also verified that the number of subjects was appropriate in view of the theoretical saturation point.

Interviews were taped and later transcribed and a discourse analysis was carried out by means of grounded theory techniques [18]. We used the notion of Discourse Analysis from Critical Social Psychology, which states that language is the basis of the social construction of reality [19]

The analysis was performed with the backing of the Atlas-ti program (v.4.2), independently for each group (cases, controls or husbands), and we based it on the following steps:

1. Theoretical categories were defined, with conceptual codes for organizing information.

2. Interviews were analyzed assigning conceptual codes to data segments that shared the same idea (open coding).

3. New categories with analytical capacity were indentified from obtained data.

4. Properties of each category were identified (axial coding).

5. Code families were integrated.

## Results

The following results are organized according to the sections mentioned above for interviews. Results are separated into sections corresponding to cases, controls and husbands.

### Cases

#### Subjectivity (see Table 1)

**Table 1:** Cases' views

**Subjectivity**
*"...but you know, now I see my case and I think, it would have been thousands of times better if I had stopped feeling embarrassed about it and got rid of all those silly hang-ups that one has, and I think that right now I would not be in this situation.". P10: case2.txt (139:145)*.
*"...and then I still neglected myself; later, I couldn't stand the pains, they were worse each time...". P 8: case1.txt (40:43)*.
*"- Did you take any tests after that? Did you visit the doctor?-No, not since the operation, until just recently, I never did anything, I never visited a doctor, nothing, until (I started feeling uncomfortable)...". P 8: case1.txt (145:151)*.
*"...and I said -no, if it isn't necessary, and since I don't have anything, I feel fine, I am not sick ...." P13: case3.txt (346:348)*.
*"...well I think, ¿don't you?, because one says – if nothing hurts, then why should I do it, isn't that so?". P18: case8.txt (185:186)*.

This category included the emotional reactions related to having cervical cancer, including the personal interpretation of signs and symptoms and whatever they elicited inside the woman.

A positive diagnosis generated different emotional reactions in women, such as fear, depression, sadness or guilt for letting time pass by while ignoring recommendations to get a PAP smear or not considering the examination important.

The depression may be so severe that, at least in one case, professional psychological help was sought. The perception of being at low risk of having cervical cancer was present prior to the women's diagnosis and it was one of the reasons for not having sought a PAP smear.

The most frequent perception of cancer was definitely negative, with fatal connotations, which made it very difficult for women to accept this diagnosis.

Symptoms interpretation played a very important role here. Symptom severity was related to their frequency and to the woman's lack of control over them, such as continuous vaginal secretions, pain and bleeding. The absence of a preventive attitude towards one's health was noticeable among these women. In fact, they believed that in the absence of symptoms, there is no need for a PAP smear.

The strongest contradiction that women express is their tendency to deny the diagnosis while they have high hopes of being cured. This attitude allowed them to face their treatment with courage. Nevertheless, not all these expectations were supported by medical prognoses, but rather by their own will to survive.

#### Family (see Table 2)

**Table 2:** Cases' views

**Family and Gender**
*"...when he heard that I had a cancer, that they were going to take out my womb, he took it very badly, perhaps from its negative side, he became distant and stopped providing any moral or financial support". P14: case4.txt (177:181)*.
*"...because, you see, I felt embarrassed, because I felt a bit more inhibited, as if I were ashamed, but later, well, when I had my children, one of them told me: -Mother, you live in the Stone Ages, you must forget about those things, don't think the doctors are going to see only you, they see a lot of people – that's how I started going for my PAP examination". P13: case3.txt (242:249)*.
*"I think that a husband can't stand a sick wife, isn't that so? Because they have to have [intercourse] relations and I feel sad sometimes because sometimes I think he is going to trade me for another woman, for a good one". P17: case7.txt (188:192)*.
*"...he said that I was no good as a woman because I never got pregnant, now with this (CC), he left me more easily". P14: case4.txt (254:260)*.

This category gathered information regarding previous experiences with cancer in the family and the degree of communication and support existing between couples and within families.

Most of the women studied women had previous experiences with cervical cancer in their family, either with their mother or a sister.

There were few women who requested a PAP smear on their own; generally they followed recommendations from their physicians and sometimes from their daughters, neighbors or friends. When they decided on their own, previous experience with cervical cancer, especially within the family, played an important motivating role.

Cancer is an event that nearly always a women experiences in solitude. They seldomly shared their experience with their family, either because they had a poor communication with their partner or because they just wanted to spare their children from suffering. Most women did not know wich other women from their own family had had PAP smears. When they wanted to share their feelings regarding their diagnosis, they usually did so with acquaintances or people of similar religious beliefs. When sharing with acquaintances, the purpose seemed to be to offer advice to other women and motivate them to undergo cancer screening. When sharing feelings with people of similar religious beliefs, women were looking for spiritual support.

For these reasons it is quite difficult to identify the kind of family support received by women, even when they declared that their relatives helped them *as much as they could*.

Facing this experience mostly on their own, women recognized an increased desire for moral support from their partners, but sometimes their reaction was to abandon them. The reason for this rupture was that a woman who was going to lose her womb, also lost the possibility of having children and becames "*useless as a woman"*.

Women expressed that they needed moral support more than anything else, and to feel protected and not alone. Women who had separated from their partners prior to the diagnosis usually received support from their children.

#### Gender (see Table 2)

This category took into consideration the perceptions, ideas and actions that reflect the social construction of the sexes as well as current socio-cultural values. Women's partners' reactions and the support they give are discussed as well as the reasons of women for avoiding cervical cancer screening. For most women, the examination itself was regarded as a particularly uncomfortable experience.

The notion of submissiveness appeared when women explained that men "must" have intercourse, and if they are not satisfied, they will look for the company of other women. Most women assumed that this behavior is completely natural and legitimate.

When faced with a confirmed diagnosis of cervical cancer, women feared abandonment by their partners. The belief standing here is that a woman ought to provide pleasure to her partner even at her own expense, and asking the physician whether it is possible to have intercourse, is a cause for embarrassment. Even among the health care staff, the sexual behavior of woman is questioned before that of her partner, as shown by some testimonies.

Another situation that is noticeable in this context is the role of women in the family's health. They look after the different family members, neglecting their own health or placing it in last place, frequently until their own health problems have become unmanageable. The invaluable compensation that these women receive is, undoubtedly, their children's love.

Motherhood is a characteristic that defines the condition of being a female. Therefore, the failure to procreate is a cause for abandonment. Aswith pregnancy and child care, health care is another responsibility assumed by women as part of their social role. This assumption is confirmed by the fact that they do not inform their husband about their cervical cancer so as not to worry him – which reflects the woman's belief that she is the only one responsible for her own health. This assumption is also held by the male partner who delegates to the woman the responsibility of following the appropriate treatment. Occasionally, women themselves justify the absence of their partners, saying that they must work in order to pay for food and for their treatment-related expenses.

The interviewed women justified their partners' behavior in two ways. Firs, at the time of the PAP smear, because they pointed out that a man does not have to be involved because health maintenance is the woman's exclusive responsibility; and second, when the diagnosis was positive for cervical cancer, women felt thaeir the partner had to become aware of the situation but his support was not an obligation given the severity of the problem.

Tolerance towards their partners, despite the lack of affection and communication in the relationship, seems to be accepted as a woman's obligation.

Sometimes, the partner's lack of affection is the reason for the woman's poor disposition, yet she does not dare admit it. Faced with the possibility of a diagnosis, women expressed a double social burden: the disclosure of a socially unacceptable behavior in the couple (promiscuity) and the ressponsability of being a women (gender role), which results in a sense of duty even if they are reproached by the others.

### Health services

The category included information about the quality of health care services received by women.

Women graded the attention received as good and expressed the same opinion about the information received from the physicians and health workers, yet these answers appear to be very limited and women remain with many doubts.

Some women stated that the doctor skipped examination (while this is the kind of action expected by them) during the first consultation, and neither told them what they had, but simply prescribed some medication.

### Controls

#### Subjectivity (see Table 3)

**Table 3:** Controls' views

**Subjectivity, Family, Gender and Health Services**
*"-You told me that your mother died of a CC; when she was sick, did you have a PAP smear? Were you worried?, did you want to know more about it?* -*No, I never thought I was going to have that, I never thought it.". P12: control1.txt (108:114)*.
*"...it seems to be more the responsibility of us women, first because it is our body, it seems more like the responsibility of women, doesn't it? (...) to worry about it (...) I think that the responsibility is one's own more than the partner's.". P18: control7.txt (221:229)*.
*"...whenever he comes, he is angry because I don't want to be with him [sexually]. He even tells me that it is because I have another [man], and I get very frightened of being with him...". P20: Control9.txt (112:114)*.
*"no, they haven't told me. I took it to the doctor and no, the actual truth, perhaps the doctor doesn't have the time, and one wants to know the actual truth about what is happening inside her, because, well, there are times that the doctors don't, don't tell us this, I mean, their point of view about where the infection came from or what the infection is, nothing..." P.15: control14.txt (118:1)*

In spite of not having a positive diagnosis, women in the control group developed a certain fear, which is fostered by the experience of having someone close to them with cervical cancer. Guilt is a feeling the controls share with the cases, generally because, their loved ones have to go through such an unpleasant experience.

The controls talked about the need for the cases' husbands to support their wives instead of keeping distant or, even worse to abandon them.

The cases close to them, especially when they were relatives, fostered feelings of sadness, pain and depression. This pain turns to fear when accompanied by the frequently mentioned notion of death.

Women in the control group often left the issue of survival up to God. Yet along with this idea, there was also the notion that scientific advances can prevent death, even though cervical cancer is seen as an incurable disease. Underlying both notions is the concept of a miracle, mixed with a form of denial.

Despite their fear, these women still perceived themselves as being at low risk of cervical cancer, a perception unaffected by living in close contact with cervical cancer cases and expressed as not taking preventive actions. These women used health care services for family consultations, but did not undergo cervical screening on their own.

#### Family (see Table 3)

Daughters are the family members that play the most important role in recommending and encouraging their mothers to participate in cervical screening.

Women in the control group admitted to have problems and misunderstandings with their husbands, besides the issue of whether or not they should seek a PAP smear. For example, men accepted more openly to have extramarital intercourses that occasionally caused them to feel guilty, and they reckoned on palliatives, such as bringing medication to their wives to cure infections that they believed were caused by their extramarital behavior.

#### Gender (see Table 3)

In general, women expressed a feeling of abandonement, they perceived they partners as estranged and not at all concerned about them. As in the cases, the controls were also embarrased by the fact that their bodies would be seen during the examination, thus revealing the prejudice that female integrity is closely linked to a sense of shame.

#### Health services (see Table 3)

In general, women expected a more complete explanation from their doctors; when told that they had a "mild infection", their anxiety lessened but their wish to learn more still remained.

### Hunsbands

#### Subjectivity (see Table 4)

**Table 4:** Husbands' views

**Subjectivity, Familiy, Gender and Health Services**
*"I hope they operate and take it out, because what else can be done? You know, one doesn't know the truth, what can I say?, what is to be done? Only the doctor knows how remove it by operating or with medication, I guess, but I don't think so, isn't that so? Because I guess it is too late, I think...". P14: husband2.txt (148:154)*.
*"Yes, I told her, let's go, what I am going to do, risk that something happens to you? What I am going to do with you here, and now with the children..., we have a little girl and a boy, what I am going to do alone with the children?". P14: husband2.txt (71:75)*.

The interviewed husbands mentioned that they felt frightened by their wives' symptoms. They found it alarming when symptoms such as bleeding, hemorrhage, intense pain and fainting were present. In view of the severity of the symptoms, surgery was perceived as the only possible treatment.

#### Family (see Table 4)

The interviewed men appeared willing to help their wives; some of them accompanied their wives to their examination and remained with them until after diagnosis was confirmed. It is important to point out, however, that the interviewed men were those who accompanied their wives to consultation – implying a potential source of bias. These men also mentioned that the daughters were who generally recommended that their mothers should undergo cervical screening.

#### Gender (see Table 4)

Among the reactions of the interviewed men, fear of a possible positive diagnosis of cancer was manifest. Concern over the privacy of one's body was not exclusive to women, the men also reported shame for the fact that their wives' bodies were seen.

Regarding the possible confirmation of diagnosis, the men expressed fears that had more to do with the children's care and attention than with their wives's health condition.

#### Health services (see Table 4)

The evaluation of the care received was dependent on the persistence of the symptoms. If symptomps remained after medical care was received, then the service was considered to be deficient. On the other hand, if the symptoms disappeared, health care was graded as being efficient.

## Discussion and conclusion

### Subjectivity

The subjective way in which the interviewed subjects of the present study expressed their experiences, is nothing more than the collective style constructed in regard to cancer in general. Several authors [20-22] established this relationship between subjectivity and the social construct. The conditions associated to cancer, such as impotence, denial, death, and fatality, have been observed in many countries and are referred to as "cancer phobia" [23]. Cervical cancer in particular, is closely related to sexuality.

The lack of attention and concern that women experience in relation to their own health is not coincidental. Within the Mexican culture, women have always been the procurers and not the beneficiaries of health. In this sense, to confront the reality of having cancer requires to repeatedly summon guilt and depression; guilt results from not taking early and preventive actions and depression from a seemingly inevitable and irreversible condition.

**Figure 1:**
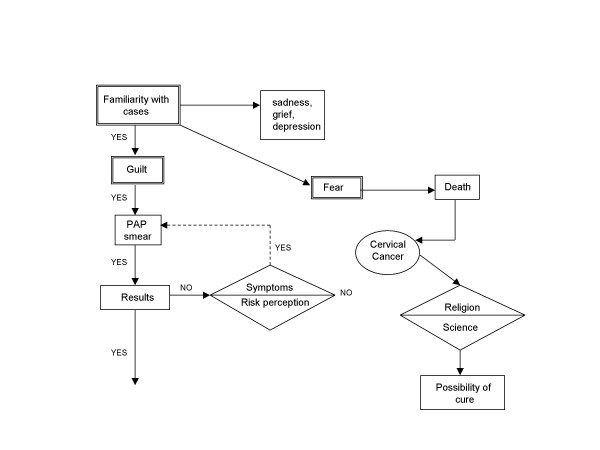
Decision process of control women.

One of our assumptions was that a close relationship with someone with a confirmed diagnosis would encourage healthy women to undergo cervical screening. However, our results did not confirm this assumption (Figure 1), instead, guilt seems to promote preventive behavior, in spite of informational campaigns. Sometimes women, motivated by guilt, seek a PAP smear but do not wait to learn its results; it wil take until they perceive a risk associated with concrete symptoms like hemorrhage, than they will seek another PAP smear.

**Figure 2:**
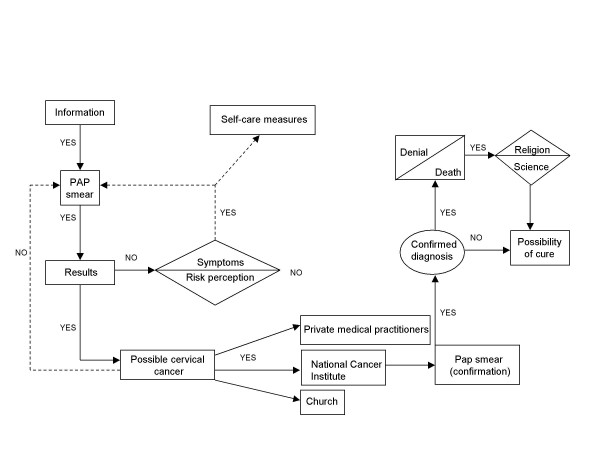
Decision process of women with cervical cancer.

As depicted in the experiential map created for women with cervical cancer, (See figure 2), information is the basic motivation that these women had to undergo an examination. When a woman does not get the PAP results and certain worrisome symptoms appear, she will probably use self-care measures like curative teas, or when symptoms are severe, she will request another PAP smear. If the results indicate cancer, women seek medical care.

In the meantime, anxiety levels rise and women find relief in religion. Facing confirmed diagnosis, the most frequent response is denial, a stage that has been identified in studies regarding grief [24], and, in this case, it could be refered to an "anticipated pain", associated with the idea of inevitable death. Again, the role of religion is important, and within the context of socialization, provides consolation in despair. Both science and religion represent possibilities of a cure for these women.

### Family

The lack of intra-family communication is clearly a problem in these families and it translates into a limited number of support networks to face situations where positive diagnosis is either suspected and/or confirmed.

The belief that cancer is exclusively the woman's responsibility probably originates in the lack of knowledge about the disease process. Women tend to believe that cervical cancer stems from their own body (not an effect of an external agent). As a result, women tend to blame themselves for the disease. These elements create psychological barriers for early detection and timely treatment.

### Gender

Gender is a category on which ideas regarding masculinity and femininity are constructed. These ideas are the basis upon which women define their lives and their view of themselves, derive their obligations and develop feelings such as guilt.

**Figure 3:**
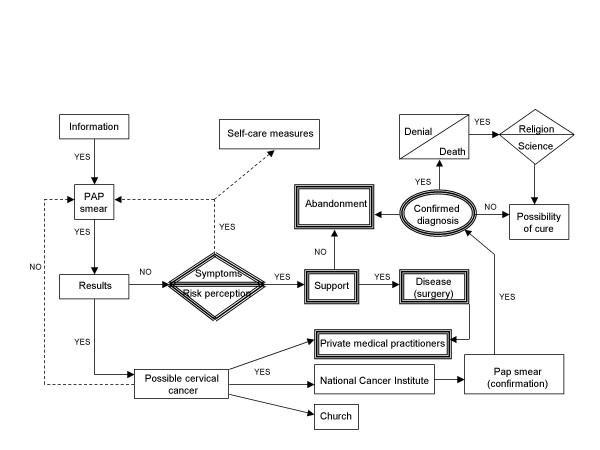
Decision process of partners of women with cervical cancer.

In the Mexican culture, masculinity is built around ideas of toughness and aggression and is associated with work [25]. Femininity is socially defined by motherhood – as is observed with the cessation of fertility and the process of menopause, which represents a tragic event for women who have assumed maternity to be basis of their identity [26]. In the present study, this emphasis on motherhood is what justifies the abandonment by the partners of women diagnosed with cancer (see Figure 3). The map reveals men's feelings and behavior (shown with double lines) in relation to women with cervical cancer. The symptoms developed by some women do worry their male partners, who see surgery as the only possible solution. Support is sometimes expressed by accompanying women to their medical appointments. However, not all men react in the same way. For some men, a woman with cervical cancer has lost the possibility of fulfilling the social role of bearing children and this constitutes a reason strong enough to leave her. In the end, abandonment confirms the lonely existence of a woman with cervical cancer.

Another conspicuous aspect revealed in this study is the concept of the body. The body is considered to be private, yet these women are the last to govern their own body, and this lack of control prevents women from going to the doctor to be examined. In the case et hand, our subjects reported that the daughters were who advised their mothers to seek a PAP smear.

Also, the lack of ownership of her own body justifies the woman's submission when faced with the extramarital behavior of her husband or partner. Hence, to confront this behavior bears a pricetag that women are not willing to pay. Such large cultural emphasis on sexuality often forces women to assume responsibility for the couple's failure.

These beliefs, which negatively affect women's health, are not easy to overcome or transform. A common effort would be needed involving the society as a whole, given that the social context provides "...the narratives we use to talk and think about ourselves" [27]. Nevertheless, it is important to become more familiar with a health-related event such as cancer. If beliefs that surround and justify certain behaviors are recognized, preventive actions can be more effective.

### Health services

From the users' point of view, public health services in Mexico are deficient because of the long waiting periods and brief consultations. This perception was confirmed in the present study. Women delay visits to the doctor because of the long waiting periods, and when they finally decide to go it is may be too late. Hence, the social, psychological and economical costs of disease rise just as Becker refers in the Health Belief Model [28].

These findings should be taken into account when planning the content of health programs which aim to modify behavior significantly.

## Competing interests

The author(s) declare that they have no competing interests.

## Authors' contributions

BEPV participated in the design and data analysis, wrote the first draft of the paper, reviewed different versions of manuscript, including final document; LLTG planned the current study and introduced the methodology, coordinated and helped to draft the manuscript; AMB participated in the development of the study and helped to draft the manuscript; MLC participated in the development of study and helped to draft the manuscript.

All authors read and approved the final manuscript.
